# Association Between the *Taq*I (rs731236 T>C) Gene Polymorphism and Dental Caries Risk: A Meta-analysis

**DOI:** 10.1089/gtmb.2020.0263

**Published:** 2021-05-17

**Authors:** Wei Lei, Haonan Tian, Yinlan Xia

**Affiliations:** ^1^ College of Stomatology, Chongqing Medical University, Chongqing, China.; ^2^ Chongqing Key Laboratory of Oral Diseases and Biomedical Sciences, Chongqing, China.; ^3^ Chongqing Municipal Key Laboratory of Oral Biomedical Engineering of Higher Education, Chongqing, China.

**Keywords:** dental caries, polymorphism, *Taq*I (rs731236 T>C), meta-analysis

## Abstract

***Objective:*** This study investigated the association of the *Taq*I (rs731236 T>C) polymorphism in the *VDR* gene with dental caries.

***Methods:*** A comprehensive literature search was performed in PubMed, Web of Science, Embase, SinoMed (the Chinese biomedical literature service system), and the Wiley Online Library. Overall comparisons and subgroup analyses based on ethnicity and the presence of dental caries in dentition were performed. Odds ratios (ORs) with 95% confidence intervals (CIs) were calculated to assess associations between gene polymorphisms and the risk of dental caries.

***Results:*** Seven articles were included in this meta-analysis. The pooled results revealed a significant association of the *Taq*I (rs731236 T>C) polymorphism with dental caries in the allele contrast model (C vs. T: OR = 1.24, 95% CI = 1.07–1.44, *I*^2^ = 42%, *p* = 0.005) and in the recessive genetic model (CC vs. TT/CT: OR = 1.38, 95% CI = 1.03–1.84, *I*^2^ = 0%, *p* = 0.03). A stratified analysis based on ethnicity revealed a significant association between the *Taq*I (rs731236 T>C) polymorphism and the risk of dental caries in Asians (C vs. T: OR = 1.28, 95% CI = 1.06–1.54, *I*^2^ = 60%, *p* = 0.009). Subgroup analysis based on the presence of dental caries in dentition found a significant association of the *Taq*I (rs731236 T>C) polymorphism with permanent tooth caries in the allele contrast model (C vs. T: OR = 1.40, 95% CI = 1.11–1.77, *I*^2^ = 76%, *p* = 0.005) and the recessive genetic model (CC vs. TT/CT: OR = 1.44, 95% CI = 1.03–2.00, *I*^2^ = 0%, *p* = 0.03).

***Conclusion:*** The results of this meta-analysis suggest that the C allele and CC genotype of the *Taq*I (rs731236 T>C) polymorphism in the *VDR* gene are associated with an increased risk of dental caries.

## Introduction

The World Health Organization (WHO) lists dental caries as the third leading major disease that seriously affects human health (after cancer and cardiovascular disease). According to the WHO, the decayed-missing-filled index decreased worldwide with the development of stomatology, but dental caries still affect the physical attributes and quality of life of 60–90% of school-aged children and most adults (Petersen, 2003). Untreated dental caries may lead to a series of problems, such as pain, malocclusion, tooth loss, and abscesses, which affect growth and development in children (Sheiham, 2006; Opal *et al.*, 2015; Zhang *et al.*, 2020). Caries is a complex and multifactorial disease caused by environmental factors, such as cariogenic bacteria, diet, oral hygiene and host factors, and genetic factors. However, some people are more likely to be affected with caries when exposed to similar environmental risk factors (Gustafsson *et al.*, 1954; Yildiz *et al.*, 2016). Increasing evidence indicates that hereditary factors may be linked to caries susceptibility (Piekoszewska-Ziętek *et al.*, 2017), and >40% of caries risk is due to genetic factors (Bretz *et al.*, 2005). Some dental caries susceptibility genes were studied. These genes are related to amelogenesis, mineralization, immune reaction, taste, and saliva (Vieira *et al.*, 2014).

Vitamin D plays an important role in tooth formation, especially in the calcification of enamel and dentin (Berdal *et al.*, 1987), and it controls the expression of classical 1,25(OH)_2_D_3_ target genes and dental proteins in the body (Papagerakis *et al.*, 2002). The teeth of vitamin D-deficient rats show obvious variations and abnormalities in morphogenesis and cell differentiation (Berdal *et al.*, 1987). A meta-analysis found that vitamin D supplementation reduced the risk of dental caries by 47% (Hujoel, 2013). The standard level of vitamin D in the blood is also related to dental caries (Schroth *et al.*, 2016). The biological functions of vitamin D are regulated through interactions with the vitamin D_3_ receptor (VDR) protein (Sutton and MacDonald, 2003). However, nutritional vitamin D deficiency or VDR gene mutations may lead to impaired function of the vitamin D pathway (Wharton and Bishop, 2003). The VDR gene is located on chromosome 12q13.11, and it contains two promoter regions, eight protein-coding exons, and six untranslated exons (Martelli *et al.*, 2014). More than 200 polymorphic sites are found in the VDR gene, and the *Taq*I (rs731236 T>C) polymorphism is the most studied variant (Qin *et al.*, 2019). *Taq*I polymorphism is characterized by a single base transition (T>C) that leads to a synonymous change at codon 352 in exon 9, which was significantly associated with several diseases, such as periodontitis, osteoporosis, and multiple sclerosis (Wang *et al.*, 2009; Zhang *et al.*, 2019; Jiang *et al.*, 2020). The T allele is associated with increased transcriptional activity, mRNA stability, and a high serum level of 1,25(OH)_2_D_3_ (Martelli *et al.*, 2014).

A considerable number of studies evaluated the association of the *Taq*I (rs731236 T>C) polymorphism with dental caries. However, their conclusions are not consistent. Therefore, we performed a meta-analysis to examine the associations between *Taq*I (rs731236 T>C) polymorphism and dental caries.

## Materials and Methods

### Search strategy

We performed a literature search in PubMed, Web of Science, Embase, SinoMed (the Chinese biomedical literature service system), and Wiley Online Library; the search was updated on August 31, 2020. The following search terms were used to identify relevant articles: “*Taq*I,” “rs731236,” “gene polymorphism,” “dental caries,” “caries,” and “decayed tooth.” We also screened the references cited in the retrieved articles to identify additional qualified studies. Gray literature and other unpublished studies were not included. The publication language was restricted to English and Chinese. We included Chinese as a publication language because of its large population. However, eligible Chinese literature was not searched. Any disagreements between the two investigators were resolved through discussions with another reviewer (Y.X.). The study was performed using the following PICO model:

Population (P): children and adults

Intervention (I): *Taq*I (rs731236 T>C) polymorphism

Comparison (C): *Taq*I (rs731236 T>C)

Outcome (O): Susceptibility to dental caries

### Inclusion and exclusion criteria

There was no population limit. The following inclusion criteria for eligible studies were used: (1) the study focused on the association of the *Taq*I (rs731236 T>C) polymorphism with the risk of caries; (2) case–control studies; and (3) studies with available data to calculate the odds ratios (ORs), 95% confidence intervals (CIs), and *p*-values. The following exclusion criteria were used: (1) reports that were not case–control studies; (2) case reports, editorials, reviews, and other nonoriginal studies; and (3) duplicate studies.

### Data extraction

Two investigators (W.L. and H.T.) separately collected the available data using a predetermined protocol. The following items were included: the surname of the first author, publication year, country, ethnicity, age, genotyping method, number of cases, number of controls, and *p*-value for Hardy–Weinberg equilibrium (HWE) in the control group.

### Methodological quality

Two reviewers (W.L. and H.T.) independently assessed the quality of the literature on the basis of the Newcastle-Ottawa Scale (NOS) criteria (Table 1). Any differences were resolved through discussion with another reviewer (Y.X.). The following items were included: (1) population selection, (2) comparability of subjects, and (3) measurement of exposure factors. NOS scores ranged from 0 to 9. A score ≥5 indicated a high-quality study, and studies with a score <5 were classified as low quality.

**Table 1.** Newcastle-Ottawa Quality Assessment Scale Case Control Studies

	(1) Is the case definition adequate?
	(a) Yes, with independent validation^☆^
	(b) Yes, for example, record linkage or based on self-reports
	(c) No description
	(2) Representativeness of the cases
	(a) Consecutive or obviously representative series of cases^☆^
Selection	(b) Potential for selection biases or not stated
	(3) Selection of controls
	(a) Community controls^☆^
	(b) Hospital controls
	(c) No description
	(4) Definition of controls
	(a) No history of disease (endpoint)^☆^
	(b) No description of source
	(1) Comparability of cases and controls on the basis of the design or analysis
Comparability	(a) Study controls for the most important factor (HWE in control group)^☆^
	(b) Study controls for any additional factor (e.g., age, gender, and smoker ratios)^☆^
	(1) Ascertainment of exposure
	(a) Secure record^☆^
	(b) Structured interview where blind to case/control status^☆^
	(c) Interview not blinded to case/control status
	(d) Written self-report or medical record only
Exposure	(e) No description
	(2) Same method of ascertainment for cases and controls
	(a) Yes^☆^
	(b) No
	(3) Nonresponse rate
	(a) Same rate for both groups^☆^
	(b) Nonrespondents described
	(c) Rate different and no designation

☆ Indicates one point.

### Statistical analysis

The *p*-value for HWE in the control group in an eligible study was assessed using the chi-squared test. *p* < 0.05 was considered a significant divergence from HWE. The associations of *Taq*I polymorphism with the risk of dental caries were evaluated using ORs with 95% CIs. We performed a *Z*-test to calculate the statistical significance of ORs, and *p* < 0.05 was deemed significant. The following models were used for *Taq*I (rs731236 T>C): allele contrast model (C vs. T), recessive genetic model (CC vs. TT/CT), dominant genetic model (CT/CC vs. TT), homozygous genetic model (CC vs. TT), and heterozygous genetic model (CT vs. TT). We performed subgroup analyses based on ethnicity and the presence of dental caries in dentition. Heterogeneity between the eligible publications was detected using Cochran's Q statistic and the *I*^2^ test. A fixed-effects model was used when the *Q* test demonstrated *p* > 0.05 or *I*^2^ < 50%. Otherwise, a random-effects model was used. All statistical analyses were performed using RevMan 5.3 software (Cochrane Collaboration) and STATA software 12.0 (STATA Corp., College Station, TX).

### Sensitivity analysis and publication bias assessment

A sensitivity analysis was performed to evaluate the stability of the results by successively removing each included study. Begg's and Egger's tests were used to detect publication bias.

## Results

### Study characteristics

**FIG. 1.**
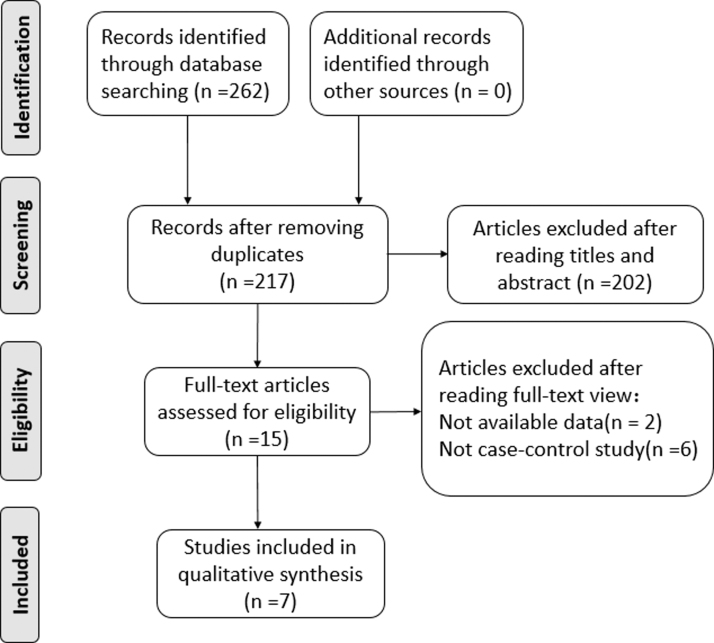
PRISMA flowchart—selection of studies for meta-analysis.

A total of 262 relevant publications were collected from the initial database search. The titles and abstracts of 15 articles were evaluated by reviewing, and 6 studies were eliminated because they were not case–control studies. After systematically reading the full text, two studies were excluded because the data were not available. Therefore, seven articles were included in this research (Hu *et al.*, 2015; Cogulu *et al.*, 2016; Izakovicova Holla *et al.* 2017; Kong *et al.*, 2017; Yu *et al.*, 2017; Qin *et al.*, 2019; Aribam *et al.*, 2020). The procedure used to retrieve the relevant publications is shown in Figure 1. The characteristics, including the surname of the first author, publication year, country, ethnicity, age, genotyping method, number of cases, number of controls, and *p*-value for HWE, are given in Table 2. The results of the quality estimation of the qualified articles are given in Table 3.

**Table 2.** Characteristics of Studies Included in the Meta-analysis

Study ID	Country	Ethnicity	Age	Genotyping method	TaqI(rs731236)		*p*-for HWE
					Case	Control	TaqI
Aribam (2020)	India	Asian	6–12 years	PCR-RFLP	60	60	0.37
Qin (2019)	China	Asian	3–5 years	TaqMan assays	304	245	0.96
Kong (2017)	China	Asian	4–7 years	PCR-RFLP	249	131	0.91
Yu (2017)	China	Asian	12 years	PCR-RFLP	200	200	0.16
Izakovicova Holla (2017)	Czech	Caucasian	13–15 years	TaqMan assays	235	153	0.11
Cogulu (2016)	Turkey	Caucasian	6–12 years	PCR-RFLP	112	38	0.32
Hu (2015)	China	Asian	30–67 years	PCR-RFLP	264	219	0.95

HWE, Hardy–Weinberg equilibrium; PCR, polymerase chain reaction; RFLP, restriction fragment length polymorphism.

**Table 3.** Newcastle-Ottawa Quality Assessment Scores for the Studies Included in the Meta-analysis

Study	Selection	Comparability	Exposure	Score
Aribam (2020)	☆☆	☆☆	☆☆	6
Qin (2019)	☆☆☆☆	☆☆	☆☆	8
Kong (2017)	☆☆☆☆	☆	☆☆	7
Yu (2017)	☆☆	☆	☆☆	5
Izakovicova Holla (2017)	☆☆☆	☆	☆☆☆	7
Cogulu (2016)	☆☆	☆☆	☆☆	6
Hu (2015)	☆☆	☆☆	☆☆	6

### Quantitative data synthesis

**FIG. 2.**
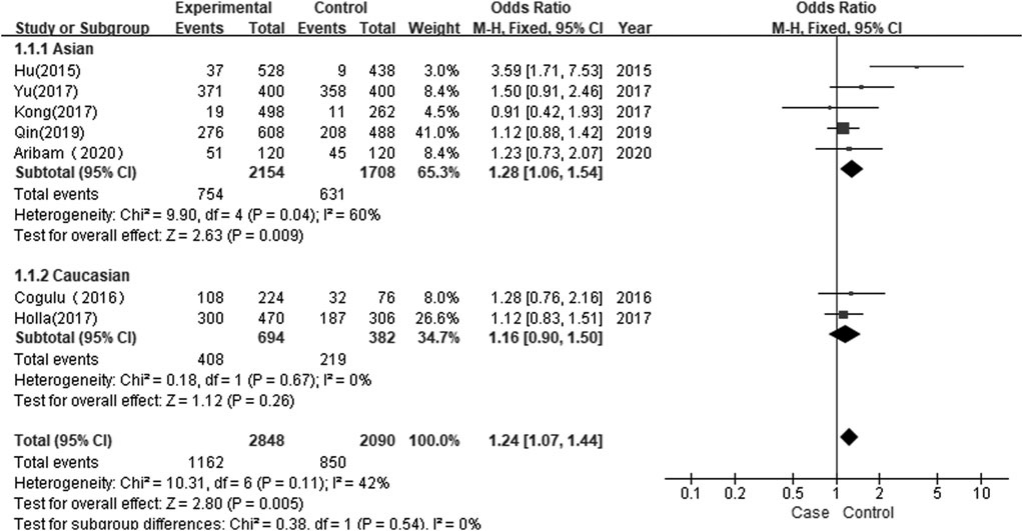
Forest plot of association between TaqI polymorphism and dental caries risk based on ethnicity (C vs. T).

**FIG. 3.**
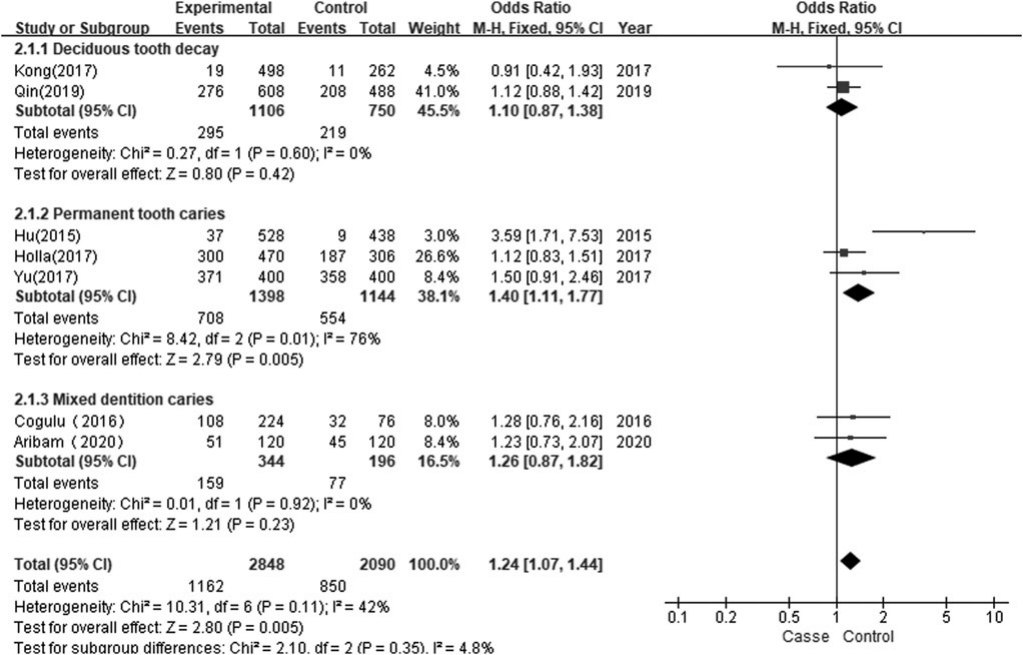
Forest plot of association between TaqI polymorphism and dental caries risk based on dental caries in dentition (C vs. T).

Seven studies with 1424 cases and 1046 controls were included to assess the association of the *Taq*I polymorphism with susceptibility to dental caries. The overall comparison found significant statistical evidence of an association between the *Taq*I polymorphism and dental caries in the allele contrast model (C vs. T: OR = 1.24, 95% CI = 1.07–1.44, *I*^2^ = 42%, *p* = 0.005) and the recessive genetic model (CC vs. TT/CT: OR = 1.38, 95% CI = 1.03–1.84, *I*^2^ = 0%, *p* = 0.03). No significant association of *Taq*I (rs731236 T>C) polymorphism with dental caries was found in the other three genetic models (dominant genetic model, CT/CC vs. TT: OR = 1.45, 95% CI = 0.96–2.19, *I*^2^ = 54%, *p* = 0.08; homozygous genetic model, CC vs. TT: OR = 0.83, 95% CI = 0.29–2.40, *I*^2^ = 75%, *p* = 0.74; heterozygous genetic model, CT vs. TT: OR = 1.41, 95% CI = 0.89–2.22, *I*^2^ = 59%, *p* = 0.14, Table 4). A stratified analysis based on ethnicity found a significant association between the *Taq*I (rs731236 T>C) polymorphism and the risk of dental caries in Asians (C vs. T: OR = 1.28, 95% CI = 1.06–1.54, *I*^2^ = 60%, *p* = 0.009; Fig. 2). The pooled results are presented in Table 4. A subgroup analysis based on the presence of dental caries in dentition revealed a significant association of the *Taq*I (rs731236 T>C) polymorphism with permanent tooth caries in the allele contrast model (C vs. T: OR = 1.40, 95% CI = 1.11–1.77, *I*^2^ = 76%, *p* = 0.005; Fig. 3) and recessive genetic model (CC vs. TT/CT: OR = 1.44, 95% CI = 1.03–2.00, *I*^2^ = 0%, *p* = 0.03). No significant association of the *Taq*I (rs731236) polymorphism with deciduous tooth decay or mixed tooth caries was found in the five genetic models (Table 4).

**Table 4.** Results of Pooled Analysis of Each Polymorphism Based on Five Genetic Models

TaqI(rs731236)		C vs. T			CT/CC vs. TT			CC vs. TT/CT			CC vs. TT			CT vs. TT		
		OR (95% CI)	*p*	*I*^2^	OR (95% CI)	*p*	*I*^2^	OR (95% CI)	*p*	*I*^2^	OR (95% CI)	*p*	*I*^2^	OR (95% CI)	*p*	*I*^2^
Total (*n*)	7	1.24 (1.07–1.44)	**0.005**	42%	1.45 (0.96–2.19)	0.08	54%	1.38 (1.03–1.84)	**0.03**	0%	0.83 (0.29–2.40)	0.74	75%	1.41 (0.89–2.22)	0.14	59%
Ethnicity																
Asian	5	1.28 (1.06–1.54)	**0.009**	60%	1.67 (0.97–2.87)	0.06	60%	1.45 (0.93–2.26)	0.10	0%	1.38 (0.55–3.50)	0.49	0%	1.67 (0.96–2.90)	0.07	60%
Caucasian	2	1.16 (0.90–1.50)	0.26	0%	1.05 (0.65–1.71)	0.84	5%	1.33 (0.91–1.95)	0.14	0%	0.62 (0.13–3.09)	0.56	87%	0.93 (0.56–1.56)	0.79	29%
Dental caries in dentition																
Deciduous decay	2	1.10 (0.87–1.38)	0.42	0%	1.39 (0.91–2.15)	0.13	43%	0.80 (0.05–12.89)	0.88	−	1.28 (0.08–21.28)	0.87	−	1.40 (0.91–2.15)	0.13	44%
Mixed caries	2	1.26 (0.87–1.82)	0.23	0%	1.37 (0.81–2.33)	0.24	0%	1.23 (0.66–2.29)	0.51	0%	1.44 (0.72–2.85)	0.30	0%	1.34 (0.75–2.40)	0.32	0%
Permanent tooth caries	3	1.40 (1.11–1.77)	**0.005**	76%	1.78 (0.41–7.73)	0.44	89%	1.44 (1.03–2.00)	**0.03**	0%	0.29 (0.16–0.53)	0.0001	−	1.65 (0.33–8.35)	0.54	90%

*p*, *p* value for test of association.

*I*^2^ is a measure of heterogeneity expressed in %.

*p* < 0.05, indicating statistical significance of bold values.

95% CI, 95% confidence interval; OR, odds ratio.

### Sensitivity analysis

**FIG. 4.**
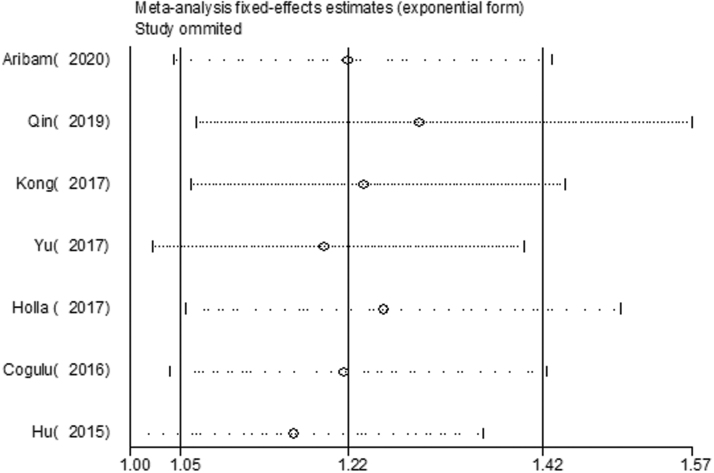
Sensitivity analysis for TaqI polymorphism and dental caries risk in an allelic model (C vs. T).

A sensitivity analysis was performed to evaluate the influence of each study by sequentially removing each included study. The significance of the pooled ORs was not affected by excluding any eligible article. Therefore, the present meta-analysis obtained stable and credible results (Fig. 4).

### Publication bias

**FIG. 5.**
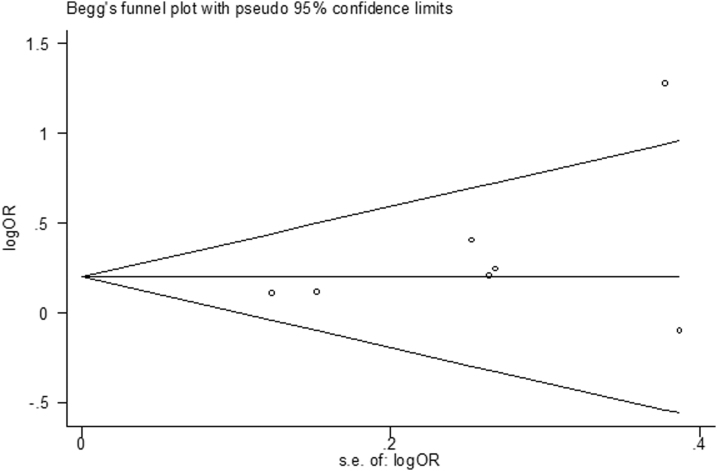
Begg's funnel plot of the TaqI polymorphism and dental caries risk in an allelic model (C vs. T).

**FIG. 6.**
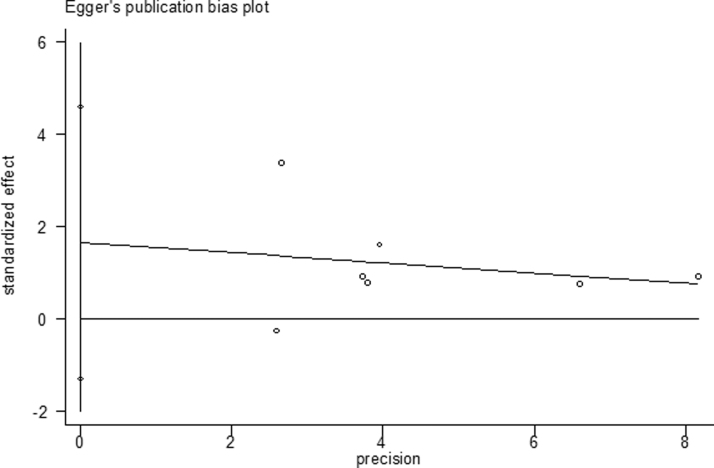
Egger's test for TaqI polymorphism and dental caries risk in an allelic model (C vs. T).

We performed Begg's test and Egger's test to detect the publication bias of each study (Figs. 5, 6). We did not detect notable publication bias for the *Taq*I (rs731236 T>C) polymorphism in any genetic model. All *p*-values from Begg's test and Egger's test are given in Table 5.

**Table 5.** The Results of Begg Test and Egger Test for Publication Bias

Polymorphisms	Comparisons	Begg test* p*-value	Egger test* p*-value
TaqI (rs731236)	C vs. T	0.368	0.208
	CC vs. TT/CT	1.000	0.988
	CT/CC vs. TT	0.221	0.188
	CC vs. TT	1.000	0.396
	CT vs. TT	1.000	0.919

## Discussion

Dental caries is a multifactorial disease that includes genetic and environmental factors. Vitamin D deficiency is related to enamel hypoplasia, which is associated with an increased risk of caries (Purvis *et al.*, 1973; Hong *et al.*, 2009). The VDR gene mediates the biological function of the vitamin D metabolite, which is related to normal enamel formation (Uitterlinden *et al.*, 2004; Hujoel, 2013). Polymorphisms of VDR in humans lead to genetic abnormalities and phenotypic diversity in tooth enamel (Zhang *et al.*, 2009). The roles of VDR gene polymorphisms in caries epidemiology attracted considerable attention in recent years. A number of studies on the associations of VDR gene polymorphisms, such as *Fok*I, *Apa*I, *Bsm*I, Cdx2, and *Taq*I, with the susceptibility to dental caries were published, and the *Taq*I (rs731236 T>C) polymorphism was the most studied variant. However, the results of these studies were inconclusive.

To our knowledge, this study is the first comprehensive study to investigate the possible association of the *Taq*I (rs731236 T>C) polymorphism with dental caries risk. Of the seven studies included in the meta-analysis of *Taq*I (rs731236 T>C), three studies indicated an association of the *Taq*I (rs731236T>C) variant with an increased risk of dental caries (Hu *et al.*, 2015; Cogulu *et al.*, 2016; Aribam *et al.*, 2020). Four studies showed that *Taq*I gene mutations could not be used as a marker for increased dental caries risk (Izakovicova Holla *et al.*, 2017; Kong *et al.*, 2017; Yu *et al.*, 2017; Qin *et al.*, 2019). Our meta-analysis results showed a significant association between the *Taq*I (rs731236 T>C) polymorphism and the risk of dental caries under the allele contrast (C vs. T) and recessive (CC vs. CT/TT) models. The ethnicity subgroup analysis showed that the *Taq*I (rs731236 T>C) polymorphism was significantly associated with the risk of dental caries in Asians but not in Caucasians. These results may reflect geographical and ethnic differences. The allele contrast (C vs. T) and recessive (CC vs. CT/TT) models found a statistically significant difference for the permanent dentition subgroup. Notably, these effects were not obtained in the deciduous dentition or mixed dentition subgroups. Bayram *et al.* (2015) and Borilova Linhartova *et al.* (2016) showed that the influence of genetic factors on enamel caries was different between deciduous and permanent teeth. Insertion/deletion (I/D) polymorphisms of angiotensin-converting enzyme may be related to dental caries in permanent teeth but not in deciduous teeth, especially in females in the Czech population (Borilova Linhartova *et al.*, 2016).

The main purpose of performing this meta-analysis was to improve the statistical power and obtain more compelling results by increasing the sample size. However, some latent shortcomings of this research cannot be ignored when interpreting the results. First, although we performed a systematic and comprehensive literature search, the number of studies that were ultimately included in the meta-analysis was limited. Second, dental caries is a multifactorial disease. However, the influence of gene–gene and gene–environment interactions was not resolved in our meta-analysis due to a lack of available data. Therefore, the potential role of the *Taq*I (rs731236 T>C) polymorphism may obscure or amplify other gene–gene/gene–environment interactions. Third, although there was no obvious publication bias according to the funnel plot and Egger's test, potential sources of bias may exist because only published studies were retrieved.

## Conclusion

The results of this study suggested that *Taq*I (rs731236 T>C) polymorphism was associated with an increased risk of dental caries. Because of the study limitations, the conclusions of this meta-analysis should be cautiously interpreted. Future studies with larger sample sizes and more ethnic groups are required to achieve more convincing and realistic statistical analyses. The underlying molecular mechanisms of the association between the *Taq*I (rs731236 T>C) polymorphism and dental caries risk should also be examined.
